# Human Milk Microbial Community Structure Is Relatively Stable and Related to Variations in Macronutrient and Micronutrient Intakes in Healthy Lactating Women

**DOI:** 10.3945/jn.117.248864

**Published:** 2017-07-19

**Authors:** Janet E Williams, Janae M Carrothers, Kimberly A Lackey, Nicola F Beatty, Mara A York, Sarah L Brooker, Bahman Shafii, William J Price, Matthew L Settles, Mark A McGuire, Michelle K McGuire

**Affiliations:** 1 Department of Animal and Veterinary Sciences,; 2 Program in Bioinformatics and Computational Biology, and; 3 Statistical Programs, College of Agricultural and Life Sciences, University of Idaho, Moscow, ID;; 4 School of Biological Sciences and; 5 Paul G. Allen School of Global Animal Health, Washington State University, Pullman, WA; and; 6 Bioinformatics Core Facility in the Genome Center, University of California, Davis, Davis, CA

**Keywords:** milk, human, microbiome, microbiota, nutrients, nutrition, BMI

## Abstract

**Background:** The human milk microbiome has been somewhat characterized, but little is known about changes over time and relations with maternal factors such as nutrient intake.

**Objective:** We sought to characterize the human milk microbiome and described associations with maternal nutrient intake, time postpartum, delivery mode, and body mass index (BMI; in kg/m^2^).

**Methods:** Milk samples (*n* = 104) and 24-h diet recalls were collected 9 times from 21 healthy lactating women from day 2 to 6 mo postpartum. Women were classified by BMI as healthy weight (<25) or overweight or obese (≥25). Bacterial taxa were characterized with the use of the high-throughput sequencing of the 16S ribosomal RNA gene.

**Results:** The milk microbiome was relatively constant over time, although there were small changes in some of the lesser-abundant genera. Relative abundances of several taxa were associated with BMI, delivery mode, and infant sex. For instance, overweight and obese mothers produced milk with a higher relative abundance of *Granulicatella* than did healthy-weight women (1.8% ± 0.6% compared with 0.4% ± 0.2%, respectively; *P* < 0.05). Relative abundances of several bacterial taxa were also associated with variations in maternal dietary intake. For example, intakes of saturated fatty acids (*r_s_* = −0.59; *P* = 0.005) and monounsaturated fatty acids (*r_s_* = −0.46; *P* = 0.036) were inversely associated with the relative abundance of *Corynebacterium*; total carbohydrates (*r_s_* = −0.54; *P* = 0.011), disaccharides (*r_s_* = −0.47; *P* = 0.031), and lactose (*r_s_* = −0.51; *P* = 0.018) were negatively associated with Firmicutes; and protein consumption was positively correlated with the relative abundance of *Gemella* (*r_s_* = 0.46; *P* = 0.037).

**Conclusions:** Factors associated with variations in the human milk microbiome are complex and may include maternal nutrient intake, maternal BMI, delivery mode, and infant sex. Future studies designed to investigate the relation between maternal nutrient intake and the milk microbiome should strive to also evaluate dietary supplement usage and analyze the collected milk for its nutrient content.

## Introduction

Human milk contains lipids, simple sugars (mainly lactose), oligosaccharides, proteins, and many other biologically active factors such as immune cells and hormones. It also contains a diverse community of bacteria (1). Culture-dependent methods have long shown the presence of various bacterial populations, such as *Staphylococcus* spp. (2) and *Lactobacillus* spp. (3, 4), but culture-independent methods have suggested a more complex bacterial community (5–7). Differences in the proportions of various genera found in milk have been reported among studies. At the phylum level, Firmicutes and Proteobacteria typically dominate (8, 9). At the genus level, *Streptococcus*, *Staphylococcus*, *Corynebacterium*, *Pseudomonas*, *Propionibacterium*, and *Bifidobacterium* are often reported as having greater relative abundances than other genera (6, 7, 9, 10).

The composition of human milk bacteria may be influenced by bacteria from the infant’s mouth (11, 12), skin, and the maternal gastrointestinal tract. Results from several studies (13–17) have provided evidence for the transfer of bacteria from the maternal gastrointestinal tract to the mammary gland through an enteromammary pathway, first hypothesized by Martín et al. (18). Thus, maternal gastrointestinal bacteria may become part of the mammary or milk microbiome, or both, and factors such as maternal nutrient intake—which is thought to directly influence the maternal gastrointestinal bacterial community—might also indirectly affect the milk microbiome.

The relation between maternal nutrient intake and gastrointestinal bacterial communities was investigated recently by Carrothers et al. (19), who provided initial evidence for associations between myriad macro- and micronutrients and maternal fecal microbial community structure during lactation. In addition, because maternal diet influences the concentration of some substances (e.g., FAs) in milk (20–23), maternal nutrient intake may indirectly help shape the bacterial community membership and structure in milk simply because of its impact on milk nutrient content. Evidence for this was provided by Kumar et al. (9), who documented multiple associations between milk FA profiles and variations in the milk microbiome. Maternal dietary intake, however, was not documented in this study.

This study was designed primarily to investigate the association between nutrient intake and milk microbiome across the first 6 mo postpartum in healthy breastfeeding women. We hypothesized that we would find correlations similar to those (between maternal dietary intake and maternal fecal microbiome) found previously by Carrothers et al. (19), although we expected the relations to be less consistent and strong. We also expected to find relations similar to those (between milk FAs and milk microbiota) described by Kumar et al. (9). Second, we investigated to what extent other variables (e.g., time postpartum, delivery mode, infant sex, maternal BMI) may also be related to variations in the human milk microbiome.

## Methods

### 

#### Subjects and study design.

This was a prospective longitudinal investigation of 21 self-reported healthy breastfeeding women. Written informed consent was obtained in accordance with procedures approved by the Washington State University and University of Idaho institutional review boards. Samples and data were collected on days 2, 5, and 10 (±1 d) and 1, 2, 3, 4, 5, and 6 mo (±1 d) postpartum. BMI (in kg/m^2^) was considered in 2 ways: prepregnancy (as reported by each subject at enrollment) and current (as measured at each sampling period). Each woman was classified as either healthy weight (<25), or overweight or obese (≥25).

#### Sample collection.

Milk samples were collected at each time point either at the subject’s home, a local hospital, Washington State University, or the University of Idaho. Women were asked to provide a full expression from one breast with the use of an Ameda-Egnell Elite pump and single-use sterile collection kit (Ameda HygieniKit). Fresh milk samples were portioned appropriately and stored immediately at −80°C or kept on ice and transported to the university, where they were portioned appropriately and frozen at −80°C within 1 h of when the samples were collected.

#### Maternal diet records.

A comprehensive quantitative 24-h dietary recall was completed for each subject at each time point. All foods and beverages (but not dietary supplements) were recorded and included in the analysis. Diet records were entered into Genesis R&D version 7.6 (ESHA Research), and energy, macronutrient intakes, and selected micronutrient intakes were estimated.

#### Extraction and amplification of bacterial DNA.

Milk samples (0.5–10 mL) were thawed on ice and centrifuged at 13,000 × *g* for 10 min at 4°C. The fat layer was carefully removed, and the supernatant was discarded. The remaining cell pellet was resuspended in 0.5 mL TE50 (10 mM Tris-HCl, 50 mM EDTA, pH 8). Samples were subjected to enzymatic lysis by adding 100 μL of a mixture containing 50 μL lysozymes (10 g/L in nuclease-free water) (Sigma-Aldrich), 6 μL mutanolysin (25 KU/mL in nuclease-free water) (Sigma-Aldrich), 3 μL lysostaphin (4000 U/mL in 20 mM sodium acetate) (Sigma-Aldrich), and 41 μL TE50 for 1 h at 37°C and then physical disruption by bead beating with 0.1-mm zirconia/silica beads (BioSpec Products) for 1 min on setting 5 with the use of a FastPrep FP120 (Qbiogene). DNA was extracted with the use of the QIAamp DNA Mini Kit (Qiagen) following the manufacturer’s protocol. TE50 (0.5 mL) was used as a negative control. Extracted DNA was eluted in 25 μL nuclease-free water and stored at −80°C until further amplification. A dual-barcoded 2-step PCR was conducted to amplify the V1–V3 hypervariable region of the bacterial 16S ribosomal RNA (rRNA) gene; a 7-fold degenerate forward primer targeting position 27 and a reverse primer targeting position 534 (positions numbered according to the *Escherichia coli* rRNA) were used (**Supplemental Table 1**).

DNA was amplified in a dedicated PCR hood. The first PCR mixture contained 5–10 μL extracted DNA, 0.05 μM target-specific primers (Integrated DNA Technologies), 1 × PCR buffer (Life Technologies), 3 mM MgCl_2_ (Life Technologies), 0.24 g BSA/L (Sigma-Aldrich), 0.2 mM deoxyribonucleotide triphosphate (Life Technologies), 0.25% DMSO, and 0.05 U AmpliTaq DNA 360 polymerase/μL (Life Technologies) in a total volume of 50 μL. PCR was conducted with the use of either an Applied Biosystems 2720, Veriti, or ProFlex model thermocycler under the following conditions: 95°C for 2 min, then 95°C for 20 s, 60°C for 30 s, and 72°C for 1 min for 20 cycles with a 0.5°C stepdown in the annealing temperature each cycle, then 95°C for 20 s, 50°C for 30 s, and 72°C for 1 min for 20 cycles, and then a final extension step of 72°C for 5 min. Samples were held at 4°C in the thermocycler until being stored at −20°C.

Products from the first PCR were evaluated for quality as described previously (19). PCR products that were deemed high quality were diluted 1:14 with nuclease-free water, and 2–4 μL were subjected to a second round of PCR in a reaction mix containing 75-nM primers with dual-index barcodes and Illumina sequencing adapters (University of Idaho IBEST Genomics Resources Core Facility), 1 × PCR buffer, 4.5 mM MgCl_2_, 0.6 g BSA/L, 0.2 mM deoxyribonucleotide triphosphate, and 0.05 U AmpliTaq DNA 360 polymerase/μL in a total volume of 20 μL. PCR was conducted with the use of an Applied Biosystems 2720 thermocycler under the following conditions: 94°C for 5 min, then 94°C for 30 s, 60°C for 45 s, and 72°C for 90 s for 20 cycles with a 0.5°C stepdown in the annealing temperature each cycle, then 94°C for 30 s, 50°C for 45 s, and 72°C for 90 s for 10 cycles, and then a final extension step of 72°C for 5 min. Samples were held at 4°C in the thermocycler until being stored at −20°C. The quality of the second PCR amplicons was evaluated by diluting the second PCR products in a 1:5 ratio with QX DNA Dilution Buffer (Qiagen) and with the use of a QIAxcel DNA screening cartridge (Qiagen). DNA was quantified with the use of the Quant-iT Picogreen dsDNA Assay kit (Invitrogen).

An appropriate volume of each amplicon was pooled to create a composite sample with an equal mass of each sample (50 ng DNA). Amplicon pools were size-selected with the use of AMPure beads (Beckman Coulter) and quantified with the use of the KAPA Biosciences Illumina library quantification kit and Applied Biosystems StepOne Plus real-time PCR system. Sequences were obtained with the use of an Illumina MiSeq v3 paired-end 300-bp protocol for 600 cycles.

#### Raw sequence analysis.

DNA sequence reads were demultiplexed and classified with the use of the custom python application dbcAmplicons (https://github.com/msettles/dbcAmplicons) to identify and assign reads by both expected barcode and primer sequences. During preprocessing, barcodes were allowed to have ≤1 mismatch (hamming distance), and primers were allowed to have ≤4 mismatches (Levenshtein distance) as long as the final 4 bases of the primer perfectly matched the target sequence. Reads were then trimmed of their primer sequence and merged into a single amplicon sequence with the use of a modified version of FLASH (https://github.com/dstreett/FLASH2) (24). The Ribosomal Database Project Bayesian classifier (25) was used to assign sequences to phylotypes. Reads were assigned to the first Ribosomal Database Project taxonomic level with a bootstrap score ≥50.

#### Longitudinal characterization of bacterial community composition.

Sequence counts were converted to relative abundance values and visualized with the use of area graphs that display the relative abundances of the most abundant taxa over time, with each color representing a different bacterial taxon. A generalized linear mixed model (GLMM) (26) assuming a β distribution and logit link was used to assess the effect of time on relative proportions of the 10 most abundant taxa. Nonmetric dimensional scaling and principal component analysis (PCA) were conducted to examine patterns among the similarities and variations, respectively, among complex bacterial community structures and other factors such as time, dietary intake of various nutrients, and birth mode.

#### Spearman rank-order correlation analysis.

Heat maps of Spearman rank-order correlation coefficients were constructed with the use of the vegan and gplots packages in R version 3.2.2 (27). These heat maps provide a 2-dimensional representation of the strength and direction of the correlations, with rectangles shaded in blue representing negative correlations and those shaded in red representing positive correlations; darker shades represent stronger associations than lighter shades. To help control for multiple comparisons, associations were deemed significant if *P* ≤ 0.01 and the Spearman sample correlation coefficient (*r_s_*) was ≤−0.3 or ≥0.3; however, because of the exploratory nature of this analysis, we also denoted weaker trends at *P* ≤ 0.05 and similar correlation coefficients. Correlations between dietary intake variables and bacterial abundances were examined with the use of both nutrient intake and bacterial abundance responses at each sampling time point and the means of the nutrient intake and bacterial abundance variables across all time points.

#### Additional inferential statistics.

Additional analyses conducted to relate selected metadata to variations in complex microbial community structures were assessed with the use of GLMMs. GLMMs assumed a β response distribution for the relative abundance response, with participant as a random effect and compound symmetric variance-covariance error structure. The dependent variables included the proportions of the 4 most abundant phyla (Firmicutes, Bacteroidetes, Actinobacteria, and Proteobacteria) and the most abundant genera (*Streptococcus*, *Staphylococcus*, *Gemella*, *Veillonella*, *Rothia*, *Lactobacillus*, *Propionibacterium*, *Corynebacterium*, *Granulicatella*, *Pseudomonas*, *Prevotella*, *Actinomyces*, *Clostridium sensu stricto*, *Neisseria*, *Bifidobacterium*, and *Haemophilus*). Independent variables were categorical metadata such as subject, parity, and birth mode. GLMM statistical analyses were carried out with the use of SAS version 9.4 (SAS Institute). *P* < 0.05 was considered significant.

## Results

### 

#### Subject description, sample disposition, and dietary intake.

Information related to basic anthropometrics and reproductive history for all 21 women at enrollment has been described previously (19). Briefly, women were 30 ± 4 y, weighed 64 ± 7 kg before pregnancy, and had a mean parity of 1.8 ± 1 children (means ± SDs).

Although milk samples were collected from most women at each time point, some women did not provide samples at certain time points, and some milk samples did not yield sufficient PCR amplicon products. In total, this resulted in 165 milk samples for which we could obtain sequencing data. In addition, because our goal was to characterize bacterial communities of milk produced by healthy women and their infants, data from 22 of the milk samples were excluded because the mother reported that she or her infant had taken antibiotics during the time since the previous sample was collected.

A total of 1,807,857 sequences were obtained following processing with a range of 19–30,900 sequences/sample. Of the 165 samples sequenced, 133 yielded >5000 sequences (range: 5180–30,900; mean ± SEM: 12,897 ± 505). After removing samples with low sequencing read counts (<5000 reads) and those associated with antibiotic use, a total of 104 milk samples were used: 5, 12, 9, 17, 15, 14, 10, 10, and 12 samples at 2, 5, and 10 d and 1, 2, 3, 4, 5, and 6 mo, respectively.

Mean dietary energy, macronutrient, and selected micronutrient intakes have also been described previously (19). In general, women consumed energy and nutrients at levels that would be expected for well-nourished lactating women (28).

#### Overall milk microbiome.

**FIGURE 1:**
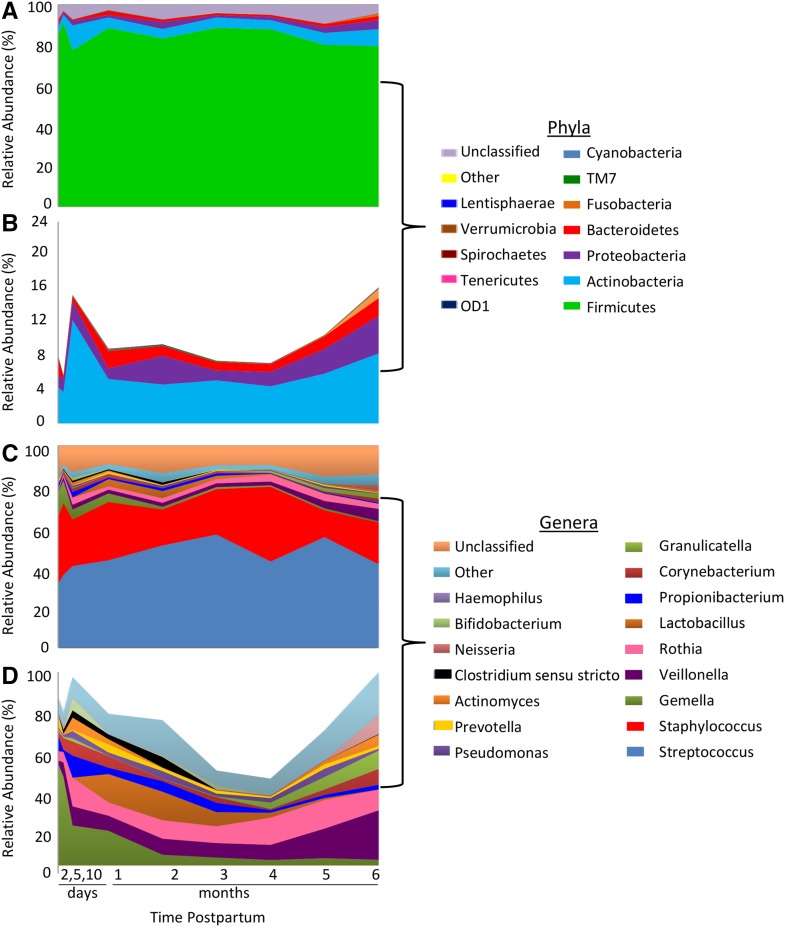
Relative abundances of the most prevalent bacterial taxa in human milk across time at the phylum level (A), enlarged view of the relative abundances of the most abundant phyla with Firmicutes omitted (B), relative abundances of the most prevalent genera across time (C), and enlarged view of the relative abundances of the most abundant genera with *Streptococcus* and *Staphylococcus* omitted (D). Colors for the different taxa are in the same order on the graph as on the key beginning at the bottom with Firmicutes for phyla and *Streptococcus* for genera. Values represent means: day 2 (*n* = 5), day 5 (*n* = 12), day 10 (*n* = 9), 1 mo (*n* = 17), 2 mo (*n* = 15), 3 mo (*n* = 14), 4 mo (*n* = 10), 5 mo (*n* = 10), and 6 mo (*n* = 12).

Relative proportions of the 4 most abundant phyla and 9 most abundant genera over all time points are found in **Table 1**. Area charts showing relative abundance of the 10 most abundant bacterial groups at each time point for phylum (12 total phyla) and genus levels (16 total genera) are shown in **Figure 1**; an enlarged view of the taxa without the dominant phyla and genera are also illustrated in this figure. In general, Firmicutes were predominant across all time points, followed by Actinobacteria, Proteobacteria, and Bacteroidetes. At the genus level, *Streptococcus* and *Staphylococcus* were predominant. *Streptococcus*, *Staphylococcus*, and *Propionibacterium* were found in all samples, whereas *Pseudomonas*, *Veillonella*, *Pilibacter*, *Gemella*, *Bacteroides*, *Prevotella*, and *Corynebacterium* were found in ≥90% of the samples. Relative abundances of the most abundant bacterial taxa in each order, class, and family are provided in **Supplemental Table 2**.

**TABLE 1:** Bacterial phyla and genera in human milk samples produced by 21 healthy women from day 2 to 6 mo postpartum^1^

Taxonomic level	Relative abundance	Range
Phylum, %		
Firmicutes	85.1 ± 1.2	22.7–97.5
Actinobacteria	5.9 ± 0.9	0.1–71.2
Proteobacteria	2.3 ± 0.3	0.1–21.3
Bacteroidetes	1.3 ± 0.3	0.1–26.7
Genus, %		
*Streptococcus*	45.2 ± 2.6	0.2–88.4
*Staphylococcus*	25.3 ± 2.6	0.1–89.1
*Gemella*	3.6 ± 0.8	0.0–51.6
*Veillonella*	2.5 ± 0.4	0.0–17.4
*Rothia*	2.4 ± 0.4	0.0–23.1
*Lactobacillus*	1.4 ± 0.6	0.0–40.7
*Propionibacterium*	1.0 ± 0.2	0.0–11.9
*Corynebacterium*	1.0 ± 0.3	0.0–18.2
*Pseudomona*	0.6 ± 0.1	0.0–4.8

1 All values are means ± SEMs unless otherwise indicated.

#### Associations between subject, time postpartum, age, parity, BMI, delivery mode, infant sex, and milk microbiome.

There was an effect of subject (*P* < 0.05) on the relative abundances of the 10 most abundant bacterial groups across the taxonomic levels, suggesting differences among women. Relations between time postpartum and relative abundances of the most abundant bacterial genera in milk are shown in **Table 2**. There was an effect of time on relative abundances of *Veillonella*, *Propionibacterium*, *Granulicatella*, and *Prevotella*. *Veillonella* increased between 4 and 6 mo (*P* = 0.01). and *Granulicatella* increased between 5 and 6 mo (*P* = 0.01). There was no effect of time postpartum on the relative abundance of any bacterial phylum.

**TABLE 2:** Top identified and unclassified genera in milk produced by 21 healthy women in the first 6 mo postpartum^1^

	Relative abundance, %								
Genus	Day 2	Day 5	Day 10	1 mo	2 mo	3 mo	4 mo	5 mo	6 mo
Samples, *n*	5	12	9	17	15	14	10	10	12
*Streptococcus*	31.5 ± 5.3	36.0 ± 6.8	40.3 ± 7.3	43.2 ± 7.4	50.5 ± 6.5	56.0 ± 8.2	42.6 ± 10.7	54.7 ± 8.4	41.3 ± 6.9
*Staphylococcus*	33.0 ± 10.7	35.7 ± 6.4	23.1 ± 8.4	28.9 ± 6.7	17.9 ± 5.8	22.6 ± 7.6	37.1 ± 12.3	13.3 ± 6.8	20.6 ± 7.1
*Gemella*	12.6 ± 6.2	11.0 ± 4.4	5.0 ± 2.3	4.3 ± 1.9	1.3 ± 0.7	1.0 ± 0.3	0.7 ± 0.3	0.9 ± 0.3	0.7 ± 0.3
*Veillonella*	0.4 ± 0.2^b^	1.8 ± 0.7^b^	2.4 ± 1.5^b^	1.9 ± 0.6^b^	2.0 ± 1.1^b^	1.8 ± 0.8^b^	1.9 ± 1.1^b^	3.7 ± 1.4^a,b^	6.1 ± 1.6^a^
*Rothia*	1.1 ± 1.1	1.3 ± 1.1	3.5 ± 2.1	1.6 ± 1.3	2.3 ± 1.1	2.1 ± 1.1	3.4 ± 1.9	3.5 ± 1.4	2.6 ± 0.6
*Lactobacillus*	0.0 ± 0.0	0.0 ± 0.0	0.0 ± 0.0	3.5 ± 2.6	3.5 ± 2.1	1.7 ± 1.1	0.6 ± 0.6	0.3 ± 0.2	0.0 ± 0.0
*Propionibacterium*	2.0 ± 1.4^a,b^	0.2 ± 0.1^d^	2.7 ± 0.8^a^	0.8 ± 0.3^c,d^	1.3 ± 0.6^b,c^	1.2 ± 0.8^b–d^	0.3 ± 0.2^d^	0.4 ± 0.2^c,d^	0.6 ± 0.4^b–d^
*Corynebacterium*	0.5 ± 0.1	1.5 ± 0.9	1.7 ± 1.2	1.5 ± 1.1	0.3 ± 0.1	0.7 ± 0.3	0.2 ± 0.1	0.7 ± 0.5	2.0 ± 1.3
*Granulicatella*	0.3 ± 0.3^b,c^	0.1 ± 0.1^b,c^	0.4 ± 0.2^b,c^	0.0 ± 0.0^c^	0.0 ± 0.0^c^	0.1 ± 0.1^c^	0.8 ± 0.7^b,c^	1.6 ± 1.0^b^	2.3 ± 0.7^a^
*Pseudomonas*	0.2 ± 0.1^b^	0.3 ± 0.1^b^	0.9 ± 0.4^a^	0.4 ± 0.1^b^	1.0 ± 0.2^a^	0.3 ± 0.1^b^	0.6 ± 0.2^a,b^	1.0 ± 0.5^a^	0.2 ± 0.1^b^
*Prevotella*	1.6 ± 1.6^a^	0.0 ± 0.0^d^	0.1 ± 0.1^d^	1.0 ± 0.9^b^	0.4 ± 0.2^c,d^	0.4 ± 0.3^b–d^	0.2 ± 0.1^c,d^	0.5 ± 0.3^b,c^	0.4 ± 0.1^c,d^
*Actinomyces*	0.0 ± 0.0	0.1 ± 0.1	1.5 ± 1.5	0.6 ± 0.5	0.1 ± 0.0	0.1 ± 0.1	0.1 ± 0.1	0.5 ± 0.2	1.4 ± 0.5
*Clostridium sensu stricto*^2^	0.1 ± 0.0	0.5 ± 0.2	0.9 ± 0.4	0.6 ± 0.3	1.3 ± 0.4	0.0 ± 0.0	0.0 ± 0.0	0.0 ± 0.0	0.1 ± 0.1
*Neisseria*^2^	0.1 ± 0.1	0.0 ± 0.0	0.0 ± 0.0	0.0 ± 0.0	0.0 ± 0.0	0.0 ± 0.0	0.0 ± 0.0	0.0 ± 0.0	2.3 ± 1.6
*Bifidobacterium*^2^	0.0 ± 0.0	0.0 ± 0.0	1.5 ± 1.5	0.0 ± 0.0	0.1 ± 0.1	0.5 ± 0.5	0.0 ± 0.0	0.0 ± 0.0	0.0 ± 0.0
*Haemophilus*^2^	0.3 ± 0.3	0.5 ± 0.3	0.1 ± 0.1	0.0 ± 0.0	0.0 ± 0.0	0.0 ± 0.0	0.0 ± 0.0	0.1 ± 0.0	0.1 ± 0.1
Other identified	18.5 ± 3.3^b,c^	12.2 ± 0.9^c^	20.2 ± 5.0^b,c^	13.7 ± 2.4^b,c^	19.6 ± 2.3^a,b^	12.5 ± 1.4^b,c^	12.5 ± 1.8^b,c^	21.3 ± 4.7^a,b^	25.8 ± 5.0^a^
Unclassified	14.5 ± 3.1	9.5 ± 0.7	13.2 ± 3.3	9.1 ± 0.9	13.6 ± 1.8	9.7 ± 1.1	9.6 ± 1.4	15.3 ± 4.6	14.2 ± 2.3

1 All values are means ± SEMs (*n* = 104) unless otherwise indicated. Values within a row not sharing a common superscript differ, *P* < 0.05. Analyses were conducted with the use of a generalized linear mixed model assuming a β distribution and logit link.

2 Estimated genera means are shown; however, no inferential analyses were carried out because of sparse response.

We found no association of mother’s age or parity with relative abundances of the overall 10 most abundant genera or the 4 most abundant phyla. There was also no association between prepregnancy BMI group, delivery mode, or infant sex and relative abundances of the most abundant bacterial phyla. However, milk produced by overweight and obese women had higher relative abundances of *Granulicatella* than that produced by healthy-weight women (1.8% ± 0.6% compared with 0.4% ± 0.2%, respectively; *P* < 0.05). There was a trend for higher *Propionibacterium* in milk from mothers who delivered via cesarean section than those delivering vaginally (1.9% ± 0.7% compared with 0.8% ± 0.2%, respectively; *P* = 0.066). Milk produced by mothers of male infants had higher *Streptococcus* and lower *Staphylococcus* than milk produced by mothers of female infants (51.7% ± 4.2% compared with 36.0% ± 5.6% and 19.2% ± 3.7% compared with 34.7% ± 6.1%, respectively; *P* < 0.05).

The PCA of the relative proportions of the overall 10 most abundant genera in milk indicated that the first-component axis accounted for 77.8% of the variations and that *Staphylococcus* and *Streptococcus* were the primary contributors to this axis. A visual examination of the PCA ordination plots (data not shown) suggested no obvious clustering patterns by time, prepregnancy BMI, infant sex, delivery mode, subject, or parity. Nonmetric multidimensional scaling analyses (data not shown) also indicated little association with these variables.

#### Associations between diet and relative abundances of single-bacterial taxa.

**FIGURE 2:**
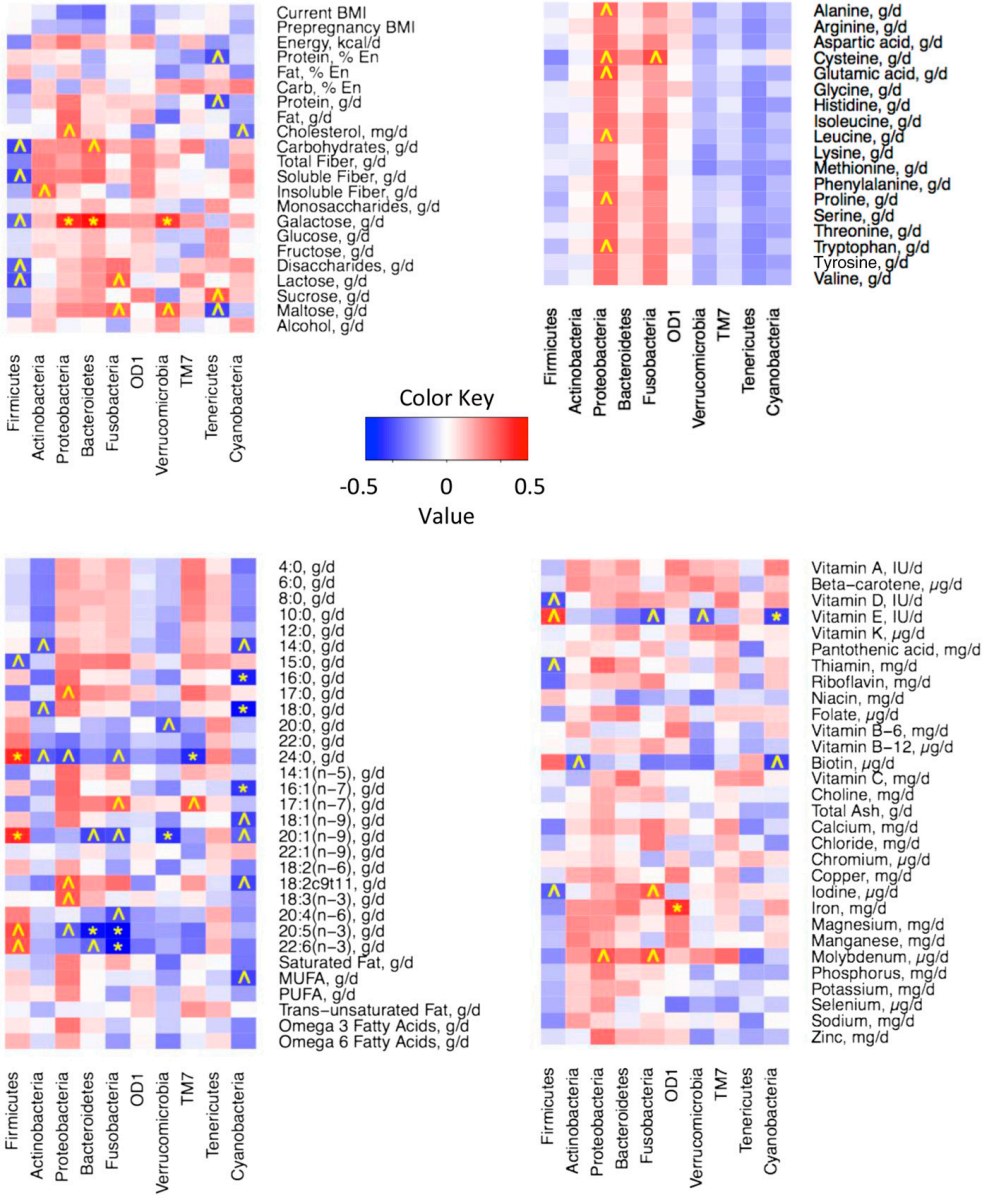
Heat maps of Spearman rank-order correlations between the relative abundance of the most abundant milk microbes at the phylum level and BMI (in kg/m^2^), energy intake, macronutrient distribution, and carbohydrate intakes (A); essential amino acid intakes (B); lipid and FA intakes (C); and vitamin and mineral intakes (D). *P ≤ 0.01 and *r_s_* ≤−0.3 or ≥0.3; ^*P* ≤ 0.05 and *r_s_* ≤−0.3 or ≥0.3. Mean variables for each woman (*n* = 21) were calculated across all time points. En, energy.

**FIGURE 3:**
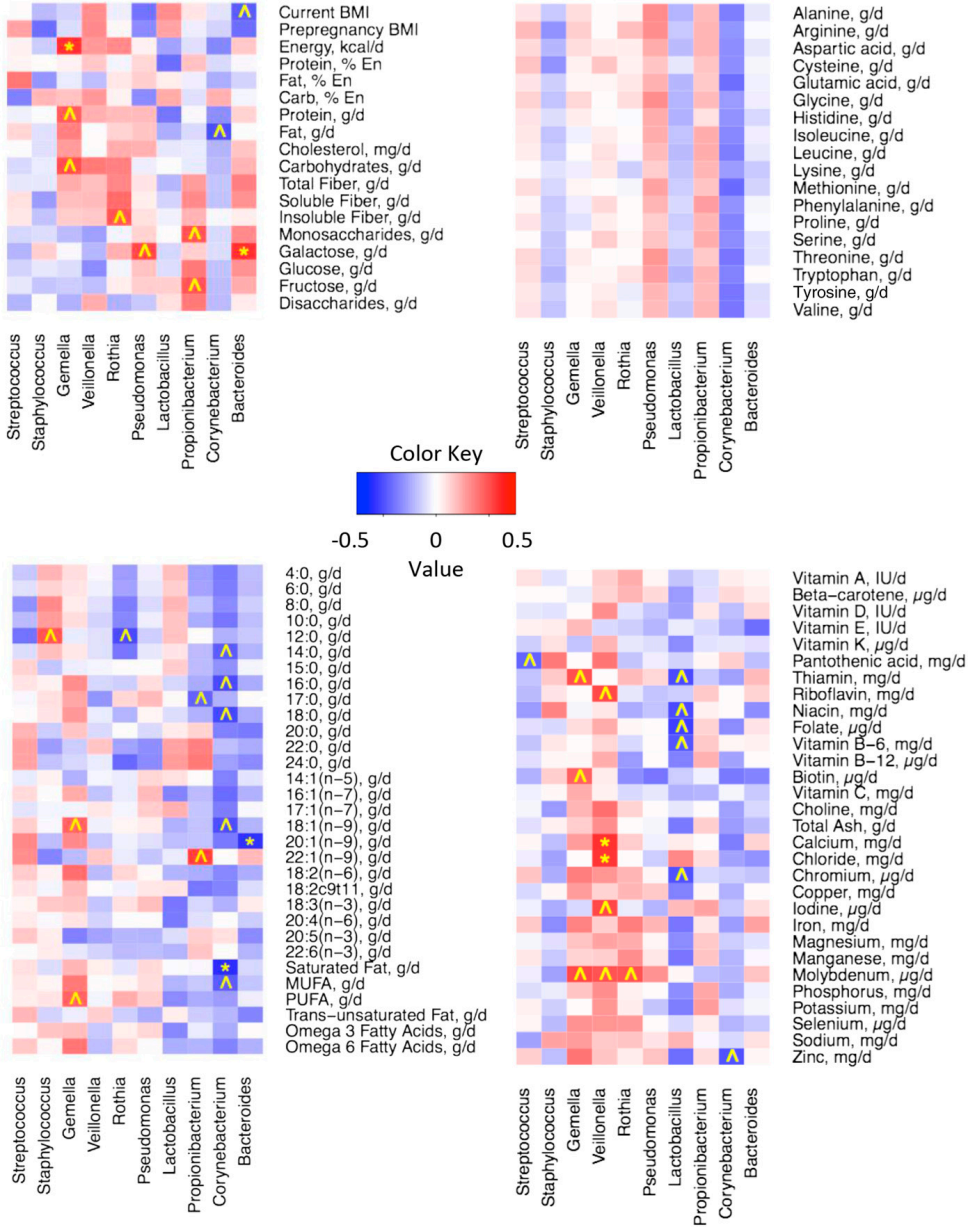
Heat maps of Spearman rank-order correlations between relative abundance of the most abundant milk microbes at the genus level and BMI (in kg/m^2^), energy intake, macronutrient distribution, and carbohydrate intakes (A); essential amino acid intakes (B); lipid and FA intakes (C); and vitamin and mineral intakes (D). *P ≤ 0.01 and *r_s_* ≤−0.3 or ≥0.3; ^*P* ≤ 0.05 and *r_s_* ≤−0.3 or ≥0.3. Mean variables for each woman (*n* = 21) were calculated across all time points. En, energy.

As described previously, we evaluated relations between nutrient intake and the milk microbiome with the use of nutrient intake and milk microbiome data at each time point (*n* = 104) and mean dietary and relative microbial abundance values across all time points (*n* = 21). When using the former approach, very few relations were apparent. However, substantially more relations were discovered when mean values were used. Heat maps illustrating these correlations are shown in **Figures 2** and **3**, and details of some of these associations are summarized in **Table 3**. Current BMI tended to be negatively correlated with *Bacteroides* (*r_s_* = −0.46; *P =* 0.037), and energy consumption was positively associated with *Gemella* (Firmicutes phylum: *r_s_* = 0.58; *P* = 0.006*)*. In general, higher SFA and MUFA intakes were related to lower *Corynebacterium* (*r_s_* = −0.59 and −0.46, respectively; *P* = 0.005 and 0.036, respectively). Total carbohydrates, total disaccharides, and lactose intakes were inversely associated with Firmicutes (*r_s_* = −0.54, −0.47, and −0.51, respectively; *P* = 0.011, 0.031, and 0.018, respectively). *Rothia* (Actinobacteria phylum) tended to be highest in milk produced by women who consumed the most insoluble fiber compared with those consuming the least insoluble fiber (*r_s_* = 0.48; *P* = 0.027), and total protein intake tended to be positively correlated with *Gemella* (*r_s_* = 0.46; *P* = 0.037). In general, greater consumption of the essential amino acids was related to an increased abundance of Proteobacteria.

**TABLE 3:** Selected significant associations between mean energy and nutrient intakes and mean percentage abundance of bacterial taxa in milk produced by 21 healthy women^1^

Diet-related variable	Bacterial taxa	*r_s_*	*P*
BMI, kg/m^2^	*Bacteroides*	−0.46	0.037
Energy, kcal/d	*Gemella*	0.58	0.006
Lipids and FAs, g/d			
SFAs	*Corynebacterium*	−0.59	0.005
MUFAs	*Corynebacterium*	−0.46	0.036
PUFAs	*Gemella*	0.44	0.045
12:0	*Staphylococcus*	0.48	0.028
*Rothia*	−0.52	0.017
14:0	Actinobacteria	−0.48	0.027
	Cyanobacteria	−0.53	0.013
	*Corynebacterium*	−0.46	0.038
15:0	Firmicutes	−0.48	0.029
16:0	*Corynebacterium*	−0.51	0.018
18:0	Actinobacteria	−0.46	0.034
	*Corynebacterium*	−0.52	0.015
20:0	Verrucomicrobia	−0.48	0.028
18:1	*Gemella*	0.44	0.045
	*Corynebacterium*	−0.50	0.022
18:2c9t11	Proteobacteria	0.48	0.026
20:1(n–9)	Firmicutes	0.60	0.004
	Bacteroidetes	−0.53	0.014
	*Bacteroides*	−0.58	0.006
20:5(n–3)	Bacteroidetes	−0.46	0.037
	Fusobacteria	−0.73	0.000
22:6(n–3)	Fusobacteria	−0.71	0.000
Carbohydrates, g/d			
Total	Firmicutes	−0.54	0.011
	Bacteroidetes	0.45	0.040
	*Gemella*	0.50	0.021
Disaccharides	Firmicutes	−0.47	0.031
Lactose	Firmicutes	−0.51	0.018
	Fusobacteria	0.46	0.035
	*Veillonella*	0.43	0.049
Maltose	Fusobacteria	0.43	0.049
	*Bacteroides*	0.58	0.006
Insoluble fiber	*Rothia*	0.48	0.027
Protein and amino acids, g/d			
Total protein	*Gemella*	0.46	0.037
Cys	Proteobacteria	0.48	0.028
Fusobacteria	0.51	0.018
Glu	Proteobacteria	0.51	0.019
Leu	Proteobacteria	0.43	0.049
Proline	Proteobacteria	0.44	0.047
Trp	Proteobacteria	0.46	0.034
Vitamins			
Pantothenic acid, mg/d	*Streptococcus*	−0.44	0.043
Riboflavin, mg/d	*Veillonella*	0.52	0.016
Thiamin, mg/d	Firmicutes	−0.44	0.049
	*Gemella*	0.50	0.022
	*Lactobacillus*	−0.51	0.017
Vitamin D, IU/d	Firmicutes	−0.50	0.022
Vitamin E, IU/d	Firmicutes	0.55	0.010
Fusobacteria	−0.51	0.018
Biotin, μg/d	Actinobacteria	−0.45	0.042
	*Gemella*	0.45	0.040
Minerals			
Calcium, mg/d	*Veillonella*	0.58	0.006
Chloride, mg/d	*Veillonella*	0.57	0.007
Iodine, μg/d	Firmicutes	−0.46	0.034
	Fusobacteria	0.47	0.030
	*Veillonella*	0.52	0.002
Iron, mg/d	OD1	0.58	0.006
Molybdenum, μg/d	*Veillonella*	0.49	0.023
	*Gemella*	0.52	0.015
	*Rothia*	0.49	0.025
Chromium, μg/d	*Lactobacillus*	−0.49	0.024
Zinc, mg/d	*Corynebacterium*	−0.49	0.023

1 The *r_s_* coefficients were determined with the use of Spearman’s rank-order correlation analysis.

We also observed multiple relations between micronutrient consumption and milk microbiome patterns. For instance, pantothenic acid intake tended to be negatively related to *Streptococcus* (*r_s_* = −0.44; *P* = 0.043). Riboflavin and calcium were both positively associated with *Veillonella* (*r_s_* = 0.52 and 0.58, respectively; *P* = 0.016 and 0.006, respectively). *Lactobacillus* also tended (*P* ≤ 0.05) to be negatively related to several micronutrients, such as thiamin (*r_s_* = −0.51), niacin (*r_s_* = −0.51), folate (*r_s_* = −0.54), vitamin B-6 (*r_s_* = −0.48), and chromium (*r_s_* = −0.49). Heat maps illustrating correlations between maternal nutrient intake and milk microbiome at the class, order, and family levels can be found in **Supplemental Figures 1–3**.

## Discussion

Results from this study reveal relatively stable microbial communities within milk produced by individual women, although differences clearly exist among women. A few bacterial populations changed over time; for instance, relative amounts of *Veillonella* increased between 4 and 6 mo, and *Granulicatella* increased between 5 and 6 mo. It is worth noting, however, that the overall 10 most abundant genera throughout the 6-mo study period did not always represent the top 10 most abundant genera at each time point, and an inspection of these individual genera indicated some minor changes over time. For this reason, future studies should more closely investigate the relative abundances of the rarer taxa.

Our data are generally similar to those previously published by others. For example, the microbial composition of colostrum we collected on day 2 postpartum was similar to that described by Obermajer et al. (29). Unlike what was reported by Cabrera-Rubio et al. (30), however, *Weisella* and *Leuconostoc* were only present in very low relative abundances (0.01% and 0.03%, respectively) in one colostrum sample we collected. In addition, *Bifidobacterium*, *Enterococcus*, and *Enterobacter* were not identified in any of our day 2 samples, and clear differences were seen between the US colostrum reported herein and that reported previously for Italian women (10). Our findings were also somewhat similar to those of Murphy et al. (31), with some notable differences, particularly at the genus level. It is unclear why our findings differed somewhat from those of others, but variations in the collection or analytical methods, or both, might have played an important role. In addition, low biomass (particularly in colostrum samples) could have limited our ability to accurately describe the scope of bacterial taxa in some samples. It is also entirely possible that genuine differences exist among populations, representing what might be an example of ecohomeorhesis, which is defined as a shift in what would be considered a “normal” homeostatic range or profile to support health in a particular ecologic or behavioral niche (32).

To our knowledge, this is the first study that describes the relations between maternal nutrient intake and the bacterial communities in women’s milk. Our data suggest that maternal diet may play a key role in determining the bacterial community in milk. Diet may exert this influence through a variety of modes, such as by altering the composition of milk components or dictating the representation of specific bacterial groups in the maternal gastrointestinal tract.

Whereas there is almost no information about the relation between maternal diet and the milk microbiome, several studies have linked diet to alterations in the gastrointestinal microbiome (33), and we recently provided evidence of associations of the maternal fecal microbiome and dietary intake by lactating women (19). Although we had hypothesized that we would find similar associations between the maternal diet and the milk microbiome, this was generally not the case—largely because the bacterial communities are quite different in these sites. However, we uncovered several other relations that are noteworthy. For instance, the maternal consumption of thiamin, niacin, folate, vitamin B-6, and chromium was negatively associated with the relative abundance of *Lactobacillus* in milk, and the consumption of several minerals was associated with the relative abundance of *Veillonella* in milk. Our finding of a generalizable positive relation between the consumption of a nutrient-rich diet and Proteobacteria in milk is of particular interest because Proteobacteria are versatile in using various carbon sources for ATP production (34). Interestingly, in our study, the relative abundance of Proteobacteria also tended to be positively related to the maternal intake of various FAs, including unsaturated [e.g., heptadecanoic (17:0)] and polyunsaturated (e.g., linoleic acid [18:3(n–3)] and rumenic acid [18:2*c*9*t*11]) categories. *Corynebacterium*, on the other hand, was inversely associated with several SFAs [e.g., myristic (14:0)] and MUFAs. Unfortunately, the FA analysis was not completed on the milk samples, so we could not evaluate whether the maternal intake of these FAs was also associated with milk FA concentration. However, Kumar et al. (9) found a multitude of relations between milk lipid profiles with the milk microbiome in samples collected in South Africa, Finland, Spain, and China. For instance, TG-bound MUFA concentration was negatively associated with Proteobacteria (*r_s_* = −0.43; *P* < 0.05), and phospholipid-bound MUFA was inversely correlated with *Lactobacillus* (*r_s_* = −0.23; *P* = 0.04). Our data, which assessed dietary FA intake rather than milk FA concentration, did not support these relations.

Nonetheless, it is intriguing to speculate that diet might affect various populations of bacteria in the mammary gland by altering the nutrient content of milk. As such, future studies regarding the milk microbiome should be designed not only to evaluate maternal nutrient intake but also the nutrient content of milk. Furthermore, in vitro studies with the use of bacteria isolated from human milk and grown on cultures containing varying amounts of these nutrients will be needed to ascertain whether the relations described herein are actually causal in nature such as we have demonstrated for human milk oligosaccharides and staphylococci (35).

Detecting relations between dietary intake and bacterial populations also relies on the accuracy of the method used to obtain dietary intake data. We found that we were only able to detect relations between dietary intake and milk bacteria when we evaluated the means of these variables over the 9 dietary assessment periods. We posit that this was largely because of the number of inaccuracies inherent in dietary data collected with the use of a single 24-h dietary recall as well as the fact that it is likely that usual (chronic) nutrient consumption is more important for shaping microbial communities than acute consumption. Thus, we urge others to consider using multiple-day dietary assessment methods in future studies as we have done herein.

Maternal diet may also be one of the factors that drove the observed differences in milk microbial communities among women with different body weights and among women with different pregnancy weight gains. Collado et al. (36) found higher *Staphylococcus* and *Akkermansia* and lower *Bifidobacterium* in milk produced by overweight women than by healthy-weight women. We did not detect any relations between current (postpartum) BMI and these genera, but we did find a negative association between *Bacteroides* and current BMI. We also did not find differences in relative abundances related to prepregnancy BMI at the phylum level. Unlike our findings, however, Kumar et al. (9) reported that Firmicutes were more abundant in milk produced by women with a higher BMI than that produced by women with a lower BMI. It is likely that the findings by Kumar et al. (9) may have been caused by a much larger range of BMI in their study’s sample populations.

We also found that only the relative abundance of *Propionibacterium* in milk from women delivering via cesarean section tended to be higher than those delivering via vaginal birth. These findings are inconsistent with those of Kumar et al. (9), Cabrera-Rubio et al. (8), and Urbaniak et al. (37). The reason for these differences among studies is not understood but, again, could be because of methodologic or genuine variation.

In addition, milk produced by the mothers of male infants had higher *Streptococcus* and lower *Staphylococcus* than milk from the mothers of female infants. These findings differ from those of Urbaniak et al. (37), who found no differences related to infant sex. To our knowledge, the importance of these findings (if any) is currently unknown.

Bacteria in milk clearly represent some of the first to which the infant is exposed and may serve as or interact with some of the first colonizers of the breastfed infant’s gastrointestinal tract. Additional controlled clinical intervention studies related to the impact of environmental choices (e.g., maternal nutrient intake, probiotic consumption, exercise) are therefore warranted to understand the basic factors that regulate the human milk microbiome. These studies should use methods appropriate for estimating dietary intake (e.g., repeated 24-h recalls or multiple prospective 24-h food records) and include milk composition analyses. If possible, they should also document the amount of bacteria present in the milk rather than just the relative abundances of the bacterial taxa and viability of the bacteria.
